# Supplementary material for: FOXM1-CD44 Signaling Is Critical for the Acquisition of Regorafenib Resistance in Human Liver Cancer Cells
Source: Int J Mol Sci. 2022 Jul 14;23(14):7782. doi: 10.3390/ijms23147782 (PMC9324640; doi:10.3390/ijms23147782)
Supplement: Supplementary file 1 [file ijms-23-07782-s001.zip › ijms-1681703-supplementary.pdf]

FOXM1-CD44 Signaling Is Critical for the Acquisition of Regorafenib Resistance in Human Liver Cancer Cells

Kenly Wuputra [1,2,3,†], Pi-Jung Hsiao [4,†], Wen-Tsan Chang [5,6,7], Po-Hsuan Wu [6], Lin-Ann Chen [6,8], Jian-Wei Huang [9], Wen-Lung Su [10], Ya-Han Yang [2,3,6], Deng-Chyang Wu [2,3,11], Kazunari K. Yokoyama [1,2,3,*] and Kung-Kai Kuo [2,3,6,7,*]

1 Graduate Institute of Medicine, Kaohsiung Medical University, Kaohsiung 80708, Taiwan; kenlywu@hotmail.com
2 Regenerative Medicine and Cell Therapy Research Center, Kaohsiung Medical University, Kaohsiung 80708, Taiwan; sal9522059@yahoo.com.tw (Y.-H.Y.); dechwu@kmu.edu.tw (D.-C.W.)
3 Cell Therapy and Research Center, Kaohsiung Medical University Hospital, Kaohsiung 80756, Taiwan
4 Department of Internal Medicine, Division of Endocrinology and Metabolism, EDA Hospital, College of Medicine, I-Shou University, Kaohsiung 82445, Taiwan; pjhsiao@gmail.com
5 Center for Cancer Research, Kaohsiung Medical University, Kaohsiung 80708, Taiwan; wtchang@kmu.edu.tw
6 Division of General & Digestive Surgery, Department of Surgery, Kaohsiung Medical University Hospital, Kaohsiung 80756, Taiwan; 1050083@gap.kmu.edu.tw (P.-H.W.); linann423@hotmail.com (L.-A.C.)
7 Department of Surgery, School of Medicine, College of Medicine, Kaohsiung Medical University, Kaohsiung 80708, Taiwan
8 Department of Surgery, Ministry of Health and Welfare Pingtung Hospital, Pingtung 900027, Taiwan
9 Department of Surgery, Kaohsiung Municipal Siaogang Hospital, Kaohsiung 812, Taiwan; whalemax@hotmail.com
10 Department of Surgery, Kaohsiung Municipal Ta-Tung Hospital, Kaohsiung 80145, Taiwan; 970401@ms.kmuh.org.tw
11 Division of Gastroenterology, Department of Internal Medicine, Kaohsiung Medical University Hospital, Kaohsiung 80756, Taiwan
* Correspondence: kazu@kmu.edu.tw (K.K.Y.); kkkuo@kmu.edu.tw (K.-K.K.); Tel: +886-7312-1101 (ext. 2729) (K.Y.Y.); +886-7312-1101 (ext. 7651) (K.-K.K.); Fax: +886-7313-3849 (K.K.Y.); +886-7321-6992 (K.-K.K.)
† These authors contributed equally to this work.

1. Materials and Methods

1.1. Cells

The human hepatocellular carcinoma cell lines HepG2 and Hep3B were prepared as described previously [1]. The regorafenib-resistant HepG2_Rego_R and Hep3B_Rego_R cell lines were generated by culturing cells with regorafenib, starting at the concentration of 2 μM and increasing it up to 6 μM for at least 6 months. Subsequently, the cells were routinely maintained at the 6 μM concentration for each experiment.

1.2. Plasmid DNA, siRNA, and shRNA Transfection

Cells (5×10^6) were transiently transfected with plasmid DNAs, siRNA, and shRNA for FOXM1 using Lipofectamine 2000 (Thermo Fisher Scientific, Waltham, MA, USA) according to the manufacturer's instructions. After incubation for 48 h, the cells were collected and harvested for subsequent experiments. After shRNA transfection for 48 h, we selected the best clone for further analysis, as assessed by functional interference activities with endogenous FOXM1, which were evaluated by Western blotting and RT-qPCR (Table 1). The siRNA for FOXM1 and the

corresponding control nontarget siRNA were purchased from Horizon Discovery (Level Biotechnology, Inc., New Taipei, Taiwan; D-001810-10-05, L-009762-00-0005). shRNAs were purchased from RNAi core, Academia Sinica, Taipei, Tawan (TRCN0000273981, ASN0000000003).

1.3. Cell Viability

Cells (5×10^3) in 96-well plates were treated with the indicated concentrations of regorafenib (Stivarga, Bayer Pharma AG, Berlin, Germany) and thiostrepton (T8902, Sigma-Aldrich, St. Louis, MO, USA) for 72 h, and cell viability was examined via MTT assay (0.5 mg/mL) according to the manufacturer's instructions. The IC_{50} of drugs was also determined.

1.4. 3D-Sphere- and Colony-Formation Assays

Sphere- and colony-formation assays were performed as described previously [1]. Briefly, for sphere formation, cells were grown in serum-free Dulbecco's Modified Eagle's Medium (DMEM, Hyclone, Cytiva, Tokyo, Japan) supplemented with B27 (Invitrogen), 20 ng/mL EGF, and 20 ng/mL bFGF (ProSpec-Tany TechnoGene Ltd., Israel). Cells were then plated in 6-well ultra-low-attachment plates (Corning, Glendale, AZ, USA) and sphere formation was assessed using a microscope after 6 days of growth. For the colony-formation assay, cells were plated in a gelatin-coated dish at a density of 5×10^2 cells. Two weeks later, colonies with a diameter >2 mm were counted after staining with Giemsa staining solution (Wako Chemicals, Tokyo, Japan).

1.5. ChIP Assay

Cells were collected with a plastic policeman, fixed in 1% formaldehyde in PBS for 8 min at room temperature with rotation, 0.125 M glycine was added, and the solution was incubated at room temperature for 5 min to quench the crosslinks. The cells were harvested in cold PBS with protease inhibitors and washed three times for 5 min each at 4°C. The collected cells were lysed by pipetting the pellet with 750 μl of SDS lysis buffer (50 mM Tris-HCl pH 8.0, 10 mM EDTA, 1% SDS) with proteinase inhibitors and incubated on ice for 30 min. The cells were then sonicated used a SONICS VC50 instrument for 20 min (5s on /15s off) on ice, which sheared the DNA to an average size of 350 bp. The antibodies of interest or IgG negative control (4 μg) were added and the cells were incubated overnight. Precleared protein A/G-agarose beads 1:1 (Millipore) were added to the samples and the samples were incubated at 4°C for 2 h. The beads were washed using four following

buffers: low-salt buffer (0.1% SDS, 0.1% Triton X-100, 150 mM NaCl, 2 mM EDTA, 20 mM Tris-HCl pH 8.0); high-salt buffer (0.1% SDS, 0.1% Triton X-100, 500 mM NaCl, 2 mM EDTA, 20 mM Tris-HCl pH 8.0); IP wash buffer (0.5 M LiCl, 1% NP-40, 1% deoxycholic acid, 100 mM Tris-HCl pH 9.0); and Tris-EDTA buffer (10 mM Tris-HCl pH 8.0, 1 mM EDTA). Samples were eluted from the beads and reverse crosslinked using 0.3 M of NaCl at 65°C overnight, after which proteinase K was used to release DNA, and phenol/chloroform/isoamyl 25:24:1 was used to isolate the DNA fragments. Data were analyzed using a real-time PCR assay. (10 mM Tris-HCl pH 8.0, 1 mM EDTA). Samples were eluted from the beads and reverse crosslinked using 0.3 M of NaCl at 65°C overnight, after which proteinase K was used to release DNA, and phenol/chloroform/isoamyl 25:24:1 was used to isolate the DNA fragments. Data were analyzed using a real-time PCR assay. The antibodies used were FOXM1 (1:1000, sc-8088), and Normal IgG (1:1000). The primers for each fragment of detection are shown in **Table 1**.

1.6. Statistical Analyses

Data are presented as the mean ± SEM from triplicate experiments and additional replicates, as indicated. One-way ANOVA ($P < 0.0001$) followed by two-tailed Student's *t*-tests was used to assess statistical significance. The survival analysis was performed using the Kaplan–Meier method, and the curves were compared using the log-rank test. A *P*-value <0.05 was considered statistically significant.

References

[1] Kuo, K.K.; Lee, K.T.; Chen, K.K.; Yang, Y.H.; Lin, Y.C.; Tsai, M.H.; Wuputra, K.; Lee, Y.L.; Ku, C.C.; Miyoshi, H., et al. Positive Feedback Loop of OCT4 and c-JUN Expedites Cancer Stemness in Liver Cancer. Stem Cells. 34 (2016) 2613-2624, 10.1002/stem.2447.

[2] Kopanja, D.; Pandey, A.; Kiefer, M.; Wang, Z.; Chandan, N.; Carr, J.R.; Franks, R.; Yu, D.Y.; Guzman, G.; Maker, A.; Raychaudhuri P. Essential roles of FoxM1 in Ras-induced liver cancer progression and in cancer cells with stem cell features. J Hepatol. 2015 Aug;63(2):429-36. doi: 10.1016/j.jhep.2015.03.023.

Supplementary Materials: The following supporting information can be downloaded at: https://www.mdpi.com/article/10.3390/ijms23147782/s1.

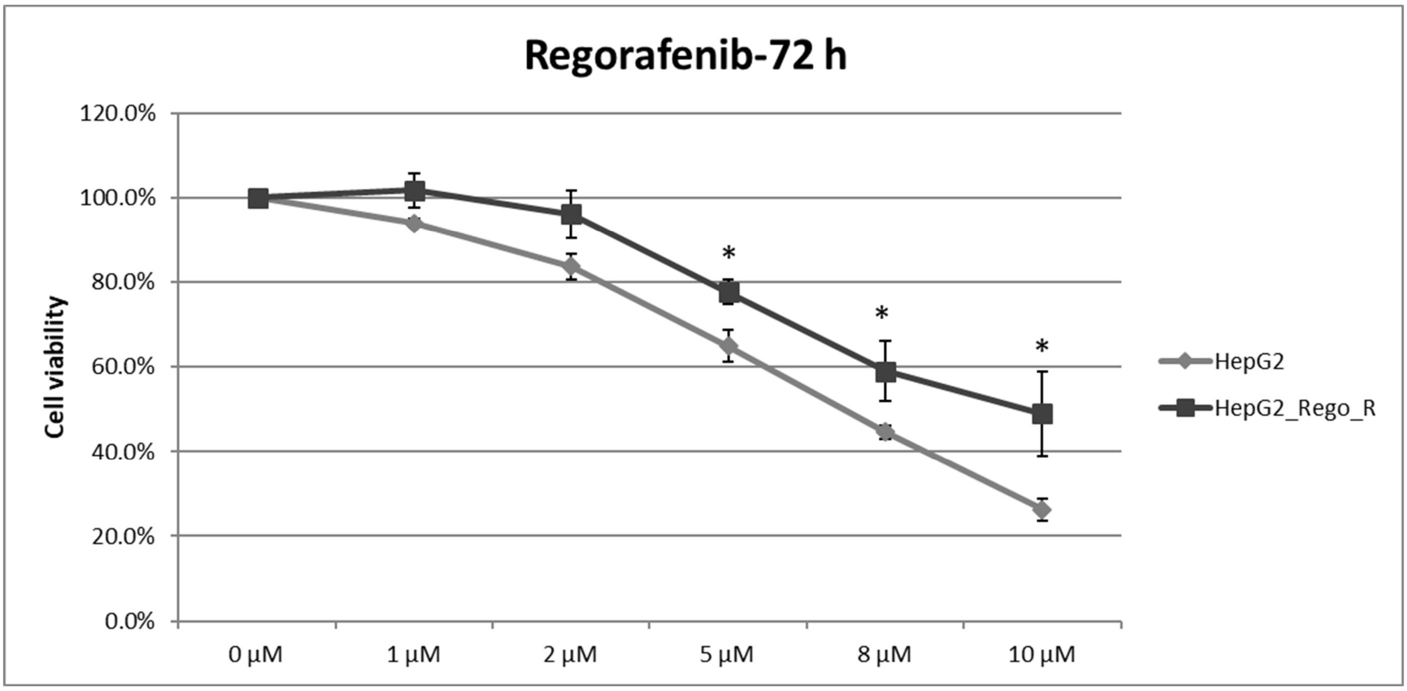

Figure S1. Effect of increasing regorafenib dose on cell viability of HepG2 and HepG2_Rego_R cells after treatment for 72 h. The cell viability was measured as described in Materials and Methods. Data were analyzed using Student's t-test (*$p < 0.05$).

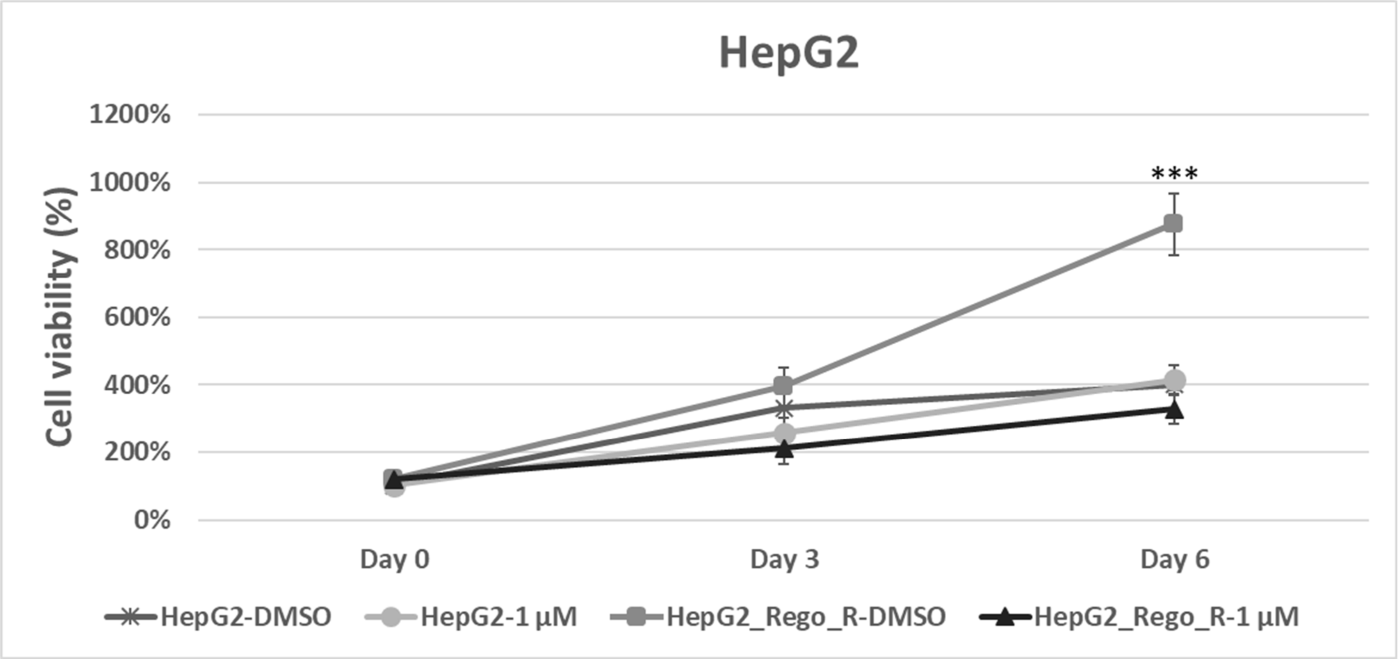

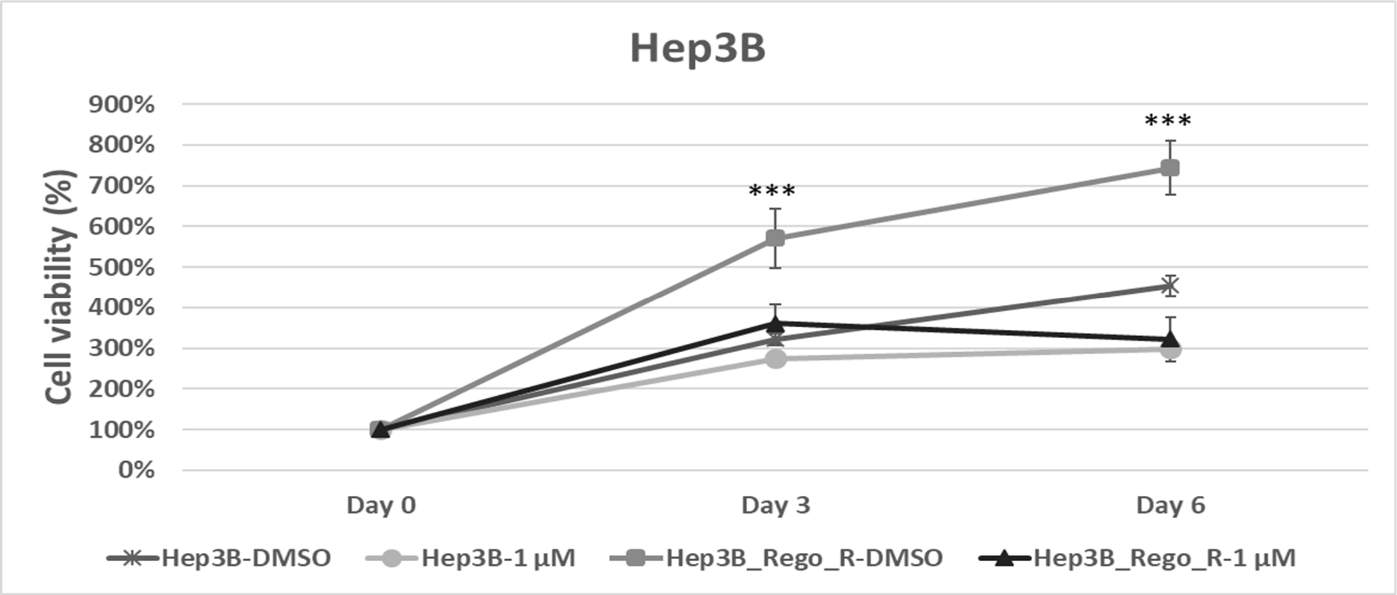

Figure S2. Comparison of cell viability of HepG2, HepG2_Rego_R, Hep3B, and Hep3B_Rego_R cell lines on treatment with 1 μM thiostrepton and 0.05% DMSO at 3 and 6 days after the treatment. The cell viability was measured as described in Materials and Methods. Data were analyzed using Student's t-test (***p< 0.001).

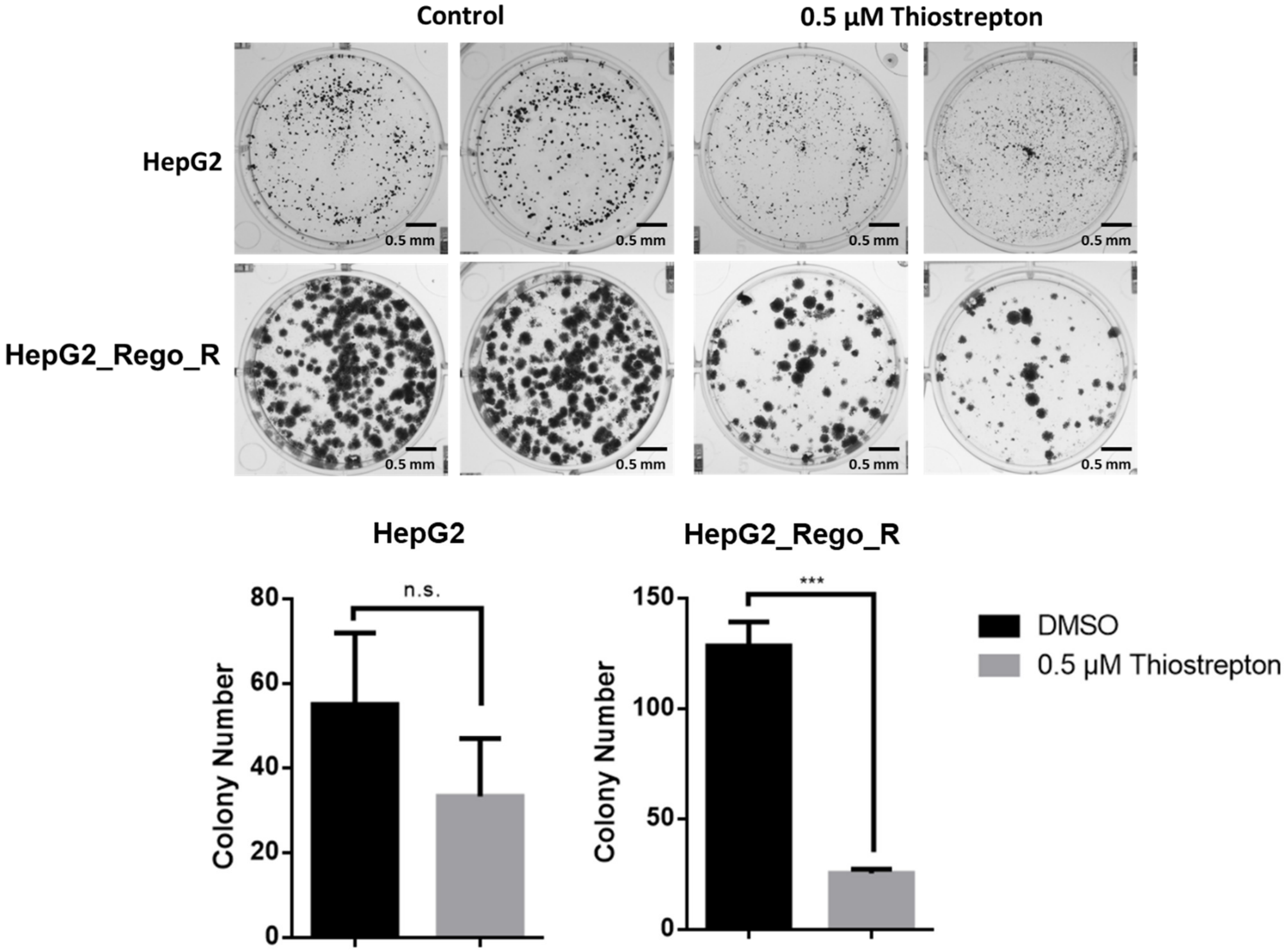

Figure S3. Comparative analysis of colonies of HepG2 and HepG2_Rego_R cells in the absence or presence of 0.5 μM thiostrepton. The colony number was calculated for each cell as described in Materials and Methods. Values represent mean ± SEM (n = 5). Data were analyzed using Student's t-test (***p< 0.001).

A

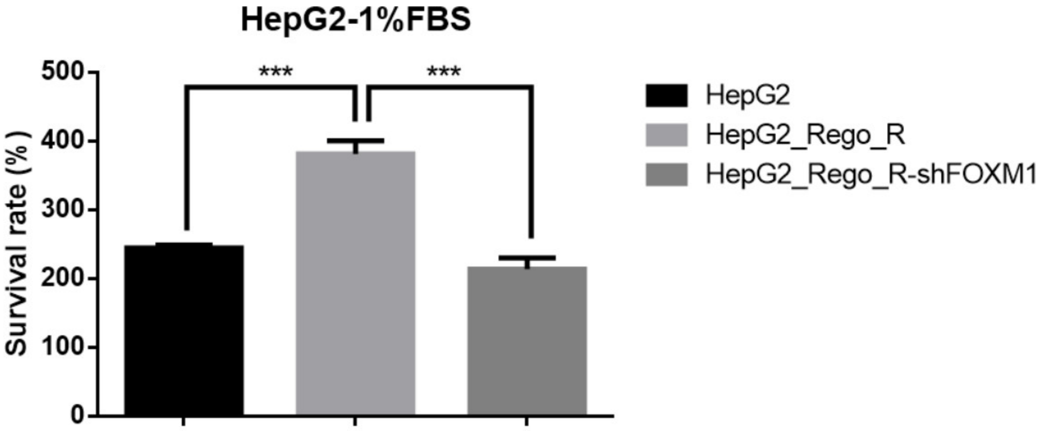

B

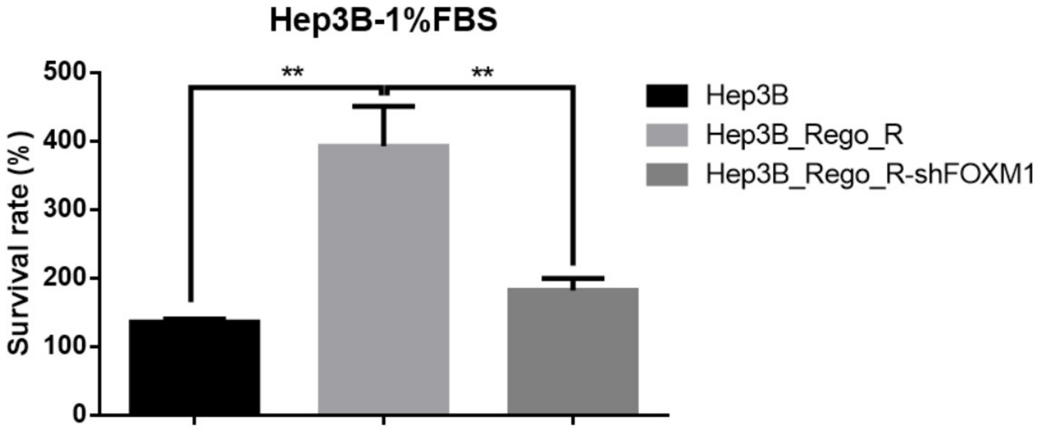

Figure S4. Effect of a lower serum concentration (1%) on cell survival in HepG2 and HepG2_Rego_R cells as well as Hep3B and Hep3B_Rego_R cells with or without shFOXM1 knockdown. The cell survival was calculated as described in Materials and Methods. Data are presented as mean ± SEM (n = 5) and analyzed using one-way ANOVA and the Tukey post hoc test (** $p < 0.01$ and *** $p < 0.001$).

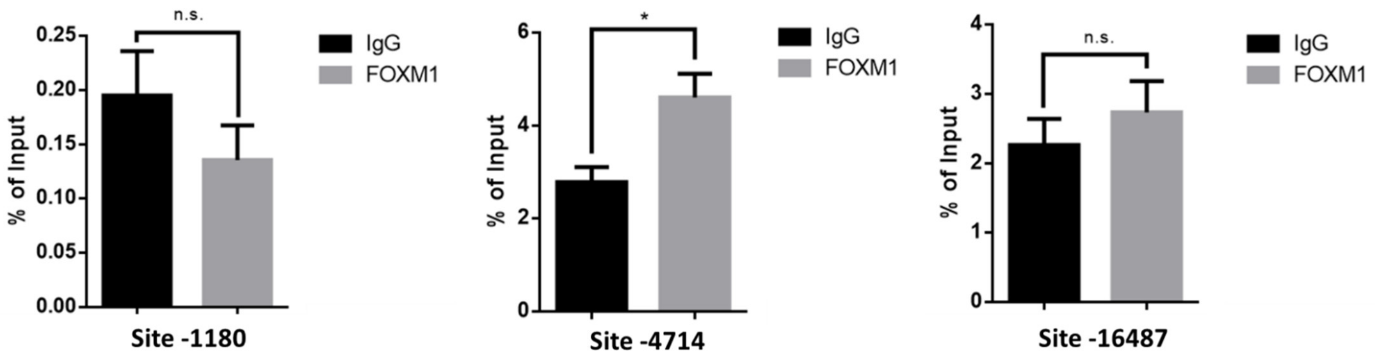

Figure S5. ChIP assay to examine the recruitment of FOXM1 to the human *CD44*-promoter region. Three putative binding sites of FOXM1 on the *CD44*-promoter region were identified based on the sequence of each site, nt 1,180, nt 4,614, and nt 16,487 [2]. ChIP assay was performed as described in Materials and Methods. Regions amplified by PCR using the specific corresponding primers (site-1180, site-4714, and site-16487) and the primers in (Table 1) *cis*-elements as indicated in HepG2. Values represent the mean ± SEM (n = 3). Data were analyzed using Student's t-test (*p < 0.05).

Figure 2B-Left

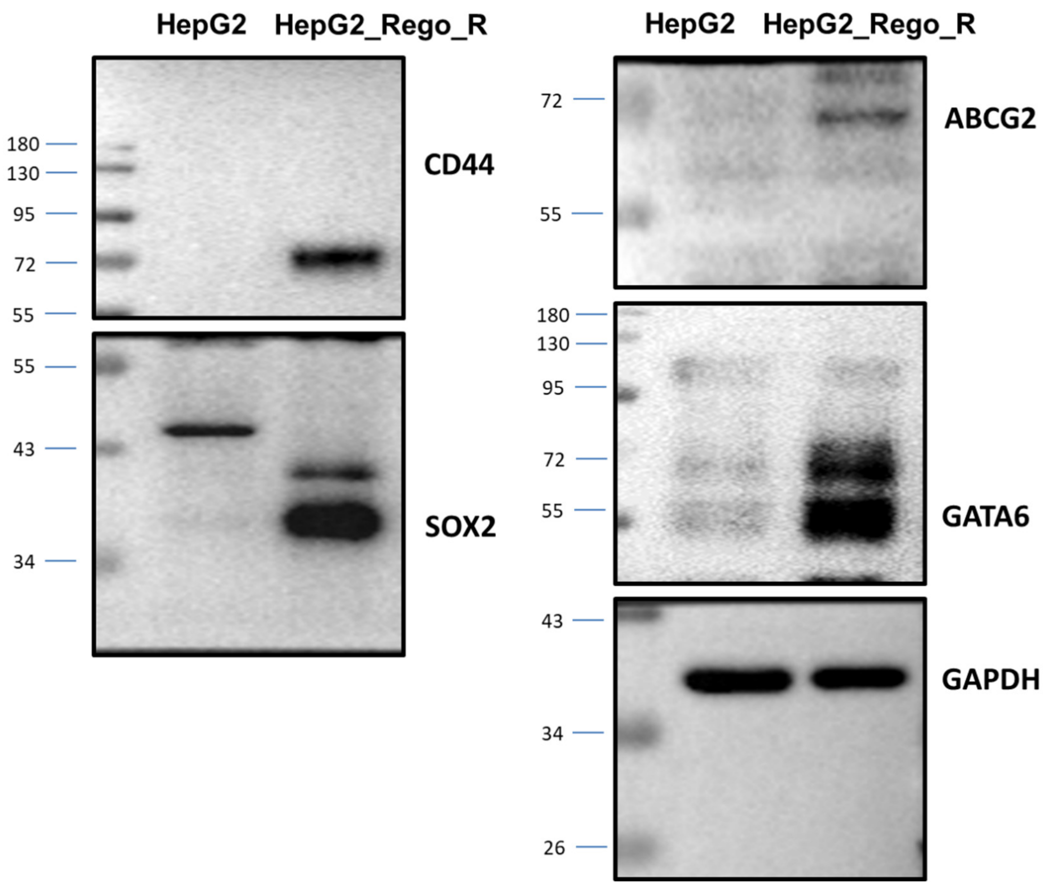

Figure 2B-Right

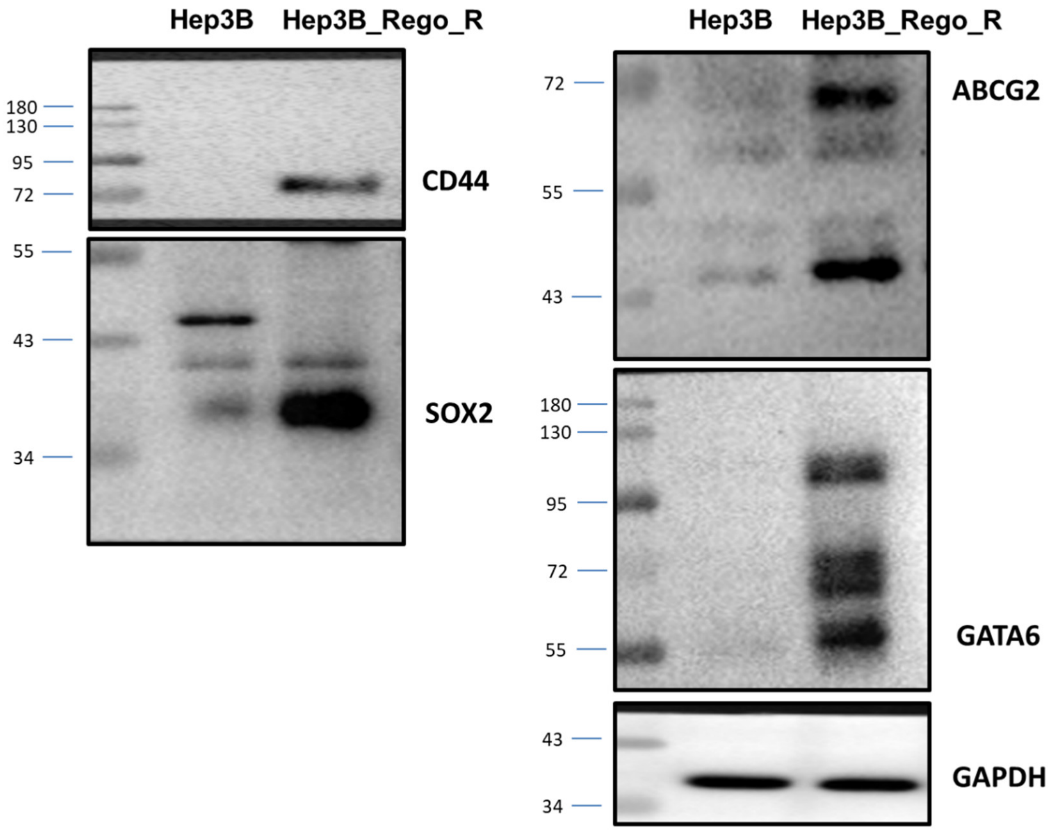

Figure 2D

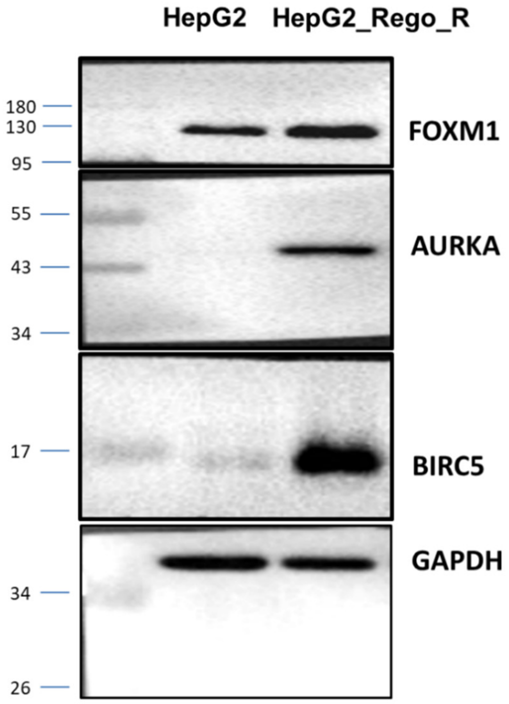

Figure 3B-Left

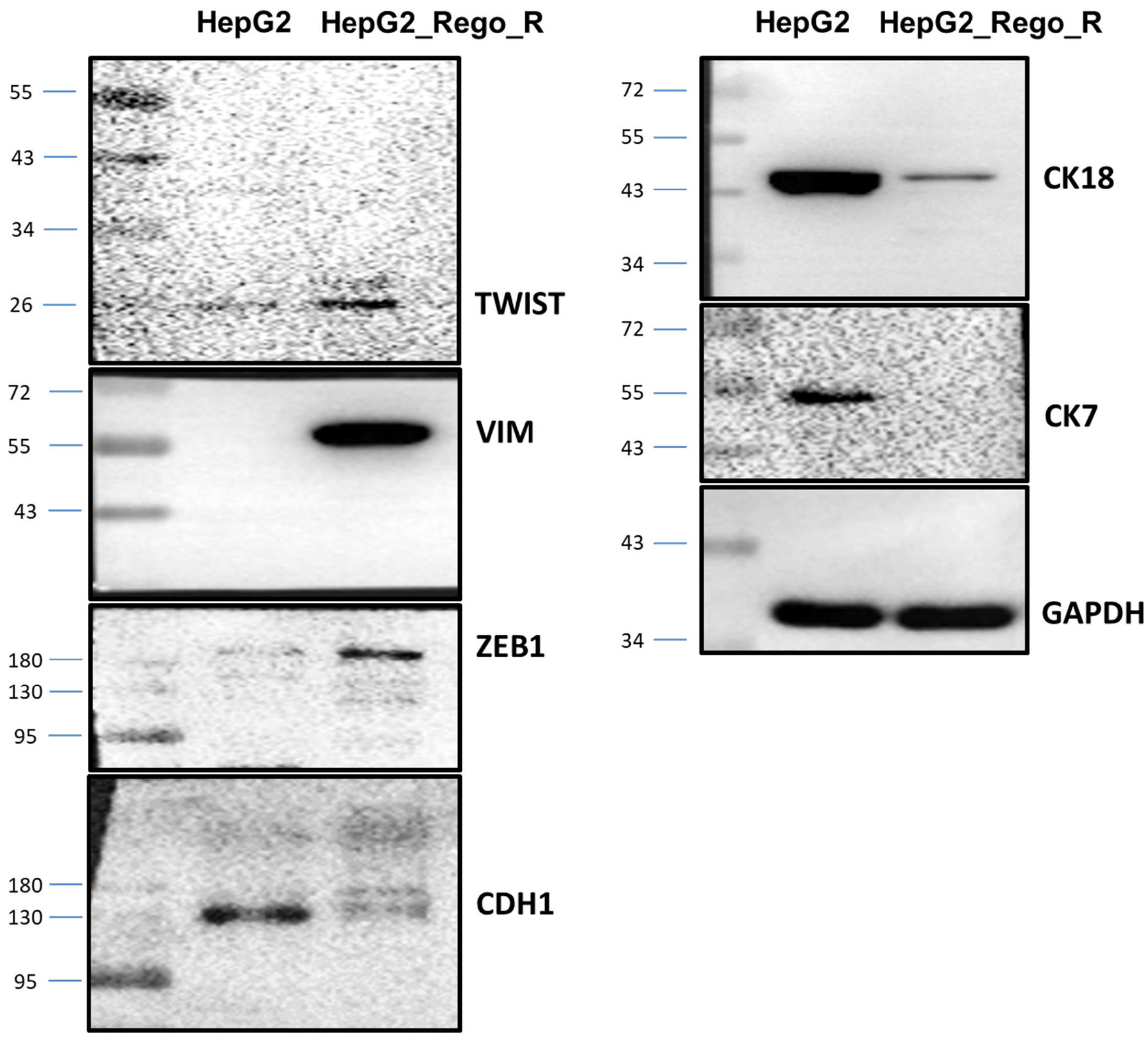

Figure 3B-Right

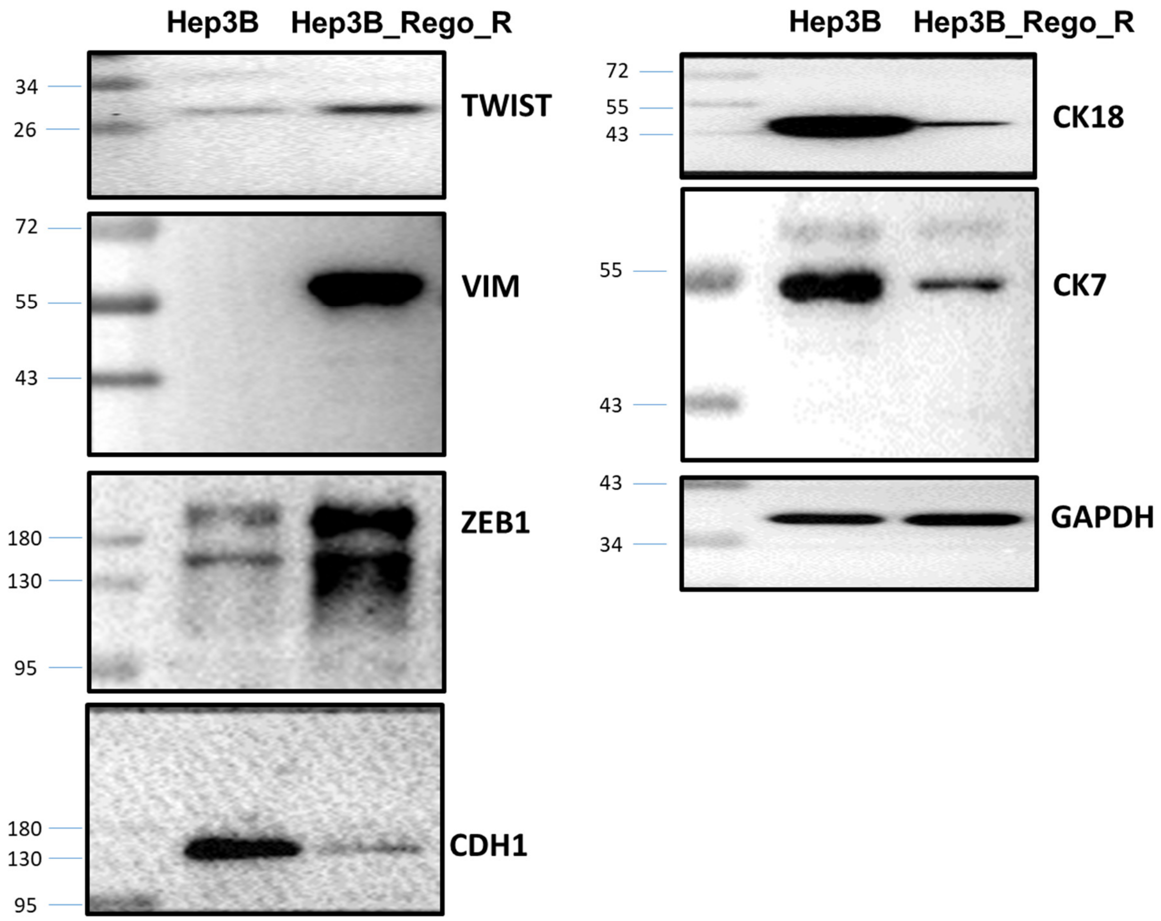

Figure 4A

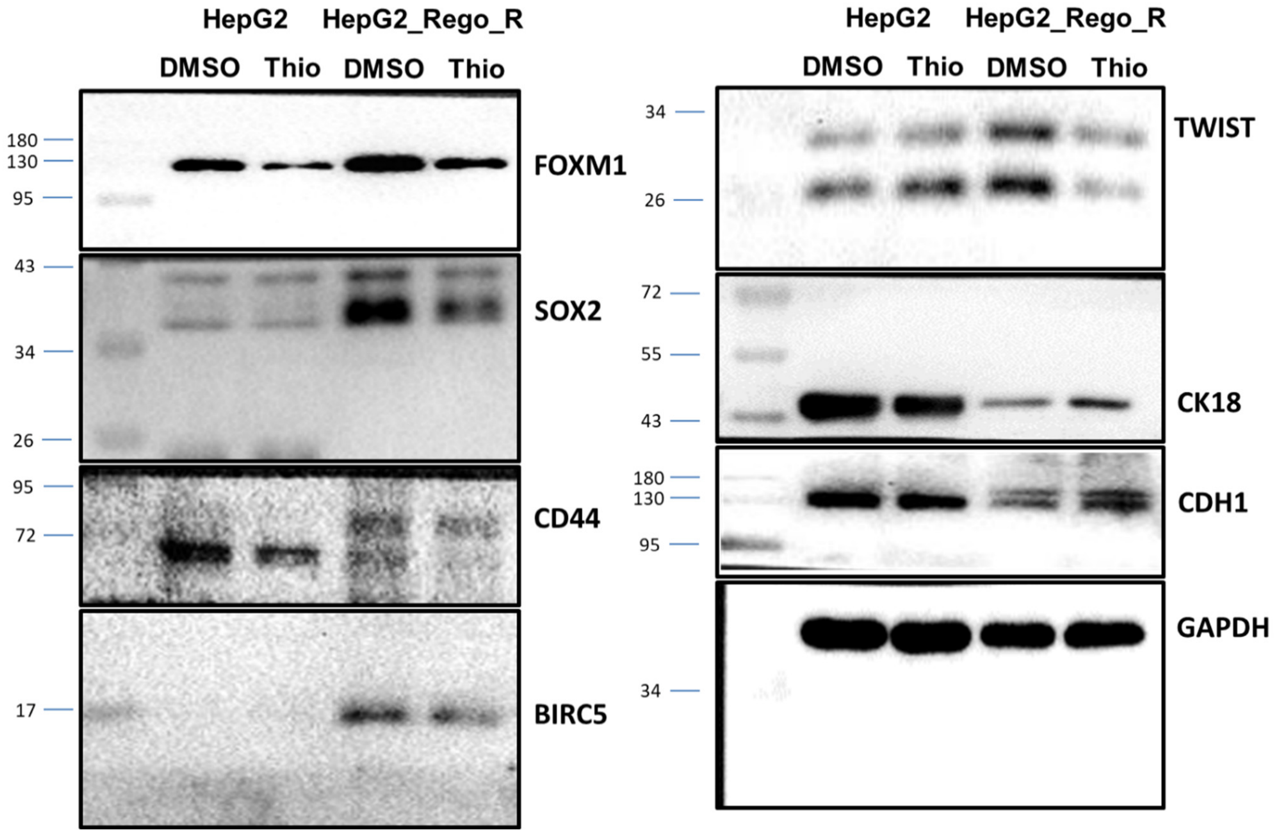

Figure 6A

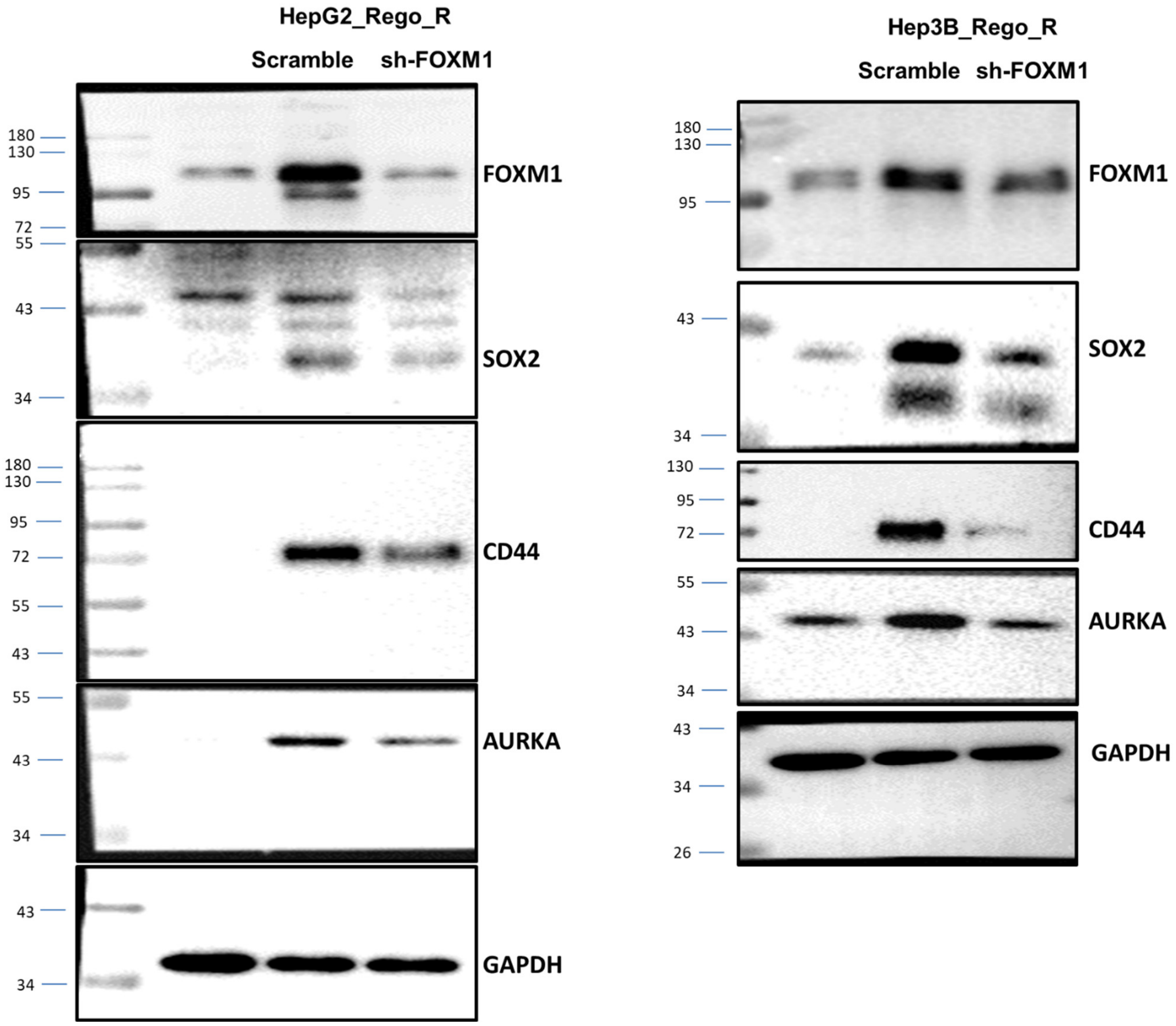

Figure S6. Full-length western blot analysis used in this study. The molecular weight markers are listed. The experiment was repeated at least three times for each protein.
